# Supplementary material for: Exam stress and the metacognitive strategies of reading in students with dyslexia: The role of motivational mechanisms and educational support
Source: PLoS One. 2023 Nov 10;18(11):e0294255. doi: 10.1371/journal.pone.0294255 (PMC10637682; doi:10.1371/journal.pone.0294255)
Supplement: Attachment — Submitted filename: Response to Reviewers.docx [file pone.0294255.s001.docx]

Dear Editor, Dear Reviewers

We are very grateful to have been given the opportunity to revise our manuscript entitled “Exam stress and the metacognitive strategies of reading in students with dyslexia: the role of motivational mechanisms and educational support” for the PLOS ONE journal (Manuscript ID: PONE-D-23-09745).

We have carefully read the guidelines and used the style templates provided. We hope that the manuscript meets PLOS ONE’s style requirements.

We would like to thank the Reviewers for all valuable comments that helped us to improve our manuscript and contributed to its quality. We have made every effort to respond to each reviewer's suggestion and we hope that we have clarified all your concerns. Below we provide detailed responses to the Reviewers' comments.

Best Regards,

Authors

**Reviewer #1**

Reviewer #1: This is an excellent study. Valid methods were used in the identification of dyslexia. The authors clearly stated the gap identified in the literature, the need for this study and its implication on the education of students with dyslexia both in Poland and the world. However, the statement on the justification for this study is not strategically positioned in the manuscript to draw readers attention, arouse their curiosity and sustain their quest to find out what new findings this new study presents or add to the existing body of literature. The identified gap could be communicated in the abstract to catch the attention of readers who focus on the abstract first. Abstract: Please briefly report on the study design, sampling approach and data analysis done in the abstract. Because this is a quantitative study, include in the results the relevant statistics (mean, SD, correlation coefficient, P-values etc.)

Authors’ response: Thank you for your appreciation. The Abstract has been modified according to the reviewer’s comments. We hope that in this form we have better exposed the information important for readers.

Reviewer #1: Methods: 1. the study design used was not mentioned

Authors’ response: Thank you for this suggestion. The information has been supplemented in accordance with the Reviewer's comment.

Reviewer #1: Methods: 2. the sampling approach used in recruiting the students from the various class or schools was/were not described.

Authors’ response: Thank you for this suggestion. The information has been supplemented in accordance with the Reviewer's comment.

Reviewer #1: Methods: 3. A Justification and adequateness of the sample size used is needed.

Authors’ response: Thank you for this suggestion. The information has been supplemented in accordance with the Reviewer's comment.

Reviewer #1: Results: The first write-up under the results be captured as data analysis.

Authors’ response: Thank you for this suggestion. We have separated the 'Data analysis' section in accordance with the reviewer's comment.

Reviewer #1: Results: Please report on the statistics in the relevant places in the main text (line 350-359 and 374-409).

Authors’ response: Thank you for this suggestion. The information has been supplemented in accordance with the Reviewer's comment.

**Reviewer #2**

Reviewer #2:  Major comment: The authors described participants for their studies, but did not show any gender differences in their results. It could be assumed that motivation and stressors in this population is considerably different between boys and girls, but no such results was mentioned.

Authors’ response: Thank you for this suggestion. Taking gender differences into account can be an important contribution to further in-depth analyzes of the material we have collected. However, in this part of the research project, we wanted to identify the mechanisms of functioning of dyslexic students in a situation of exam stress in relation to the entire study group, regardless of gender. Some identified gender differences will be presented in another manuscript.

Reviewer #2:  Minor comments: Typos, unclear sentences, and wrong prepositions in the introduction section makes reading difficult.E.g.,
1. "In fact, stress presents as one of the key challenges for educational and therapeutic practice focused on the organization of educational support" unclear sentence
2. "...and who, upon reaching higher grades of primary school have to use their often limited literacy skills in producing written assignments and passing written exams as required in the he curriculum." in the curriculum has extra articles.
3. "Tasks connected reading and writing can be seen as challenges, that trigger negative emotions and constitute potentially demanding situations, requiring educational support." Sentence is unclear.

The manuscript, specially the introduction part needs to be checked for typos and better readability.

Authors’ response: We read the manuscript carefully and made every effort to eliminate all Typos and unclear sentences.

Reviewer #2: 3. Have the authors made all data underlying the findings in their manuscript fully available? The PLOS Data policy requires authors to make all data underlying the findings described in their manuscript fully available without restriction, with rare exception (please refer to the Data Availability Statement in the manuscript PDF file). The data should be provided as part of the manuscript or its supporting information, or deposited to a public repository. For example, in addition to summary statistics, the data points behind means, medians and variance measures should be available. If there are restrictions on publicly sharing data—e.g. participant privacy or use of data from a third party—those must be specified.

Authors’ response: Data availability information is provided at the end of the manuscript.
