# Supplementary material for: Exam stress and the metacognitive strategies of reading in students with dyslexia: The role of motivational mechanisms and educational support
Source: PLoS One. 2023 Nov 10;18(11):e0294255. doi: 10.1371/journal.pone.0294255 (PMC10637682; doi:10.1371/journal.pone.0294255)
Supplement: Attachment — Submitted filename: Response to Reviewers_2.docx [file pone.0294255.s002.docx]

Dear Editor, Dear Reviewers

We are very grateful to have been given the opportunity to revise our manuscript entitled “Exam stress and the metacognitive strategies of reading in students with dyslexia: the role of motivational mechanisms and educational support” for the PLOS ONE journal (Manuscript ID: PONE-D-23-09745).

We have carefully read the guidelines and used the style templates provided. We hope that the manuscript meets PLOS ONE’s style requirements.

We would like to thank the Reviewers for all valuable comments that helped us to improve our manuscript and contributed to its quality. We have made every effort to respond to each reviewer's suggestion and we hope that we have clarified all your concerns. Below we provide detailed responses to the Reviewers' comments.

Best Regards,

Authors

**Reviewer #1: Based on the information provided by the authors on page 12, Line 276-278, it is still not clear how the authors arrived at the sample size 640. Did they use the Taro Yamane formular, Krejcie & Mogan formular or the Cochrane formular. Again with a 95% confidence interval, the margin of error or the acceptable sampling error is expected to be 5% but they quoted 4%. When you tabulate the values provided by the authors into the Yamane and Krejcie & Mogan formular, you still do not arrive at their estimated sample size (64). if these calculations were not factored into the determination of the sample size prior to the study, i suggest they discuss that as a methodological limitation in their study.**

Authors’ response: Thank you for this suggestion. There was a typo in the text. Instead of 4% it should be 5%, which has been corrected. There was a typo in the text. Instead of 4% it should be 5%, which has been corrected. Using the Taro Yamane formula (for N = 5,000,000 Polish students with dyslexia and 95% confidence interval), the minimum size of the study sample for the population of students with dyslexia in Poland should be 400 people. This condition has therefore been met. Due to the fact that it was possible to collect a larger number of respondents who met the criteria, the authors decided to perform analyzes on data from a larger sample.

**Reviewer #3: The authors have done thorough literature review on the subject matter. Below are my observations and suggestions after review**

**1. Line 33. If you are referring to your paper, I suggest “the study showed” to “studies” to avoid any comprehension issues to the readers.**

Authors’ response: Thank you for this suggestion. The change suggested by the Reviewer was made.

**2. Both British and American spellings are employed; perhaps adhering to one standard would be optimal.**

Authors’ response: Thank you for this suggestion. The change suggested by the Reviewer was made.

**3. Line 131, no citation for the findings reported**

Authors’ response: Thank you for this suggestion. The information has been supplemented in accordance with the Reviewer's comment.

**4. Full stop after the in text citation 52 and 77**

Authors’ response: Thank you for this suggestion. The change suggested by the Reviewer was made.

**5. Line 135 going “Yet, it appears that students with dyslexia have comparable insight into their own difficulties and are able to adjust their reading comprehension expectations, much like students without any specific reading difficulties. Both groups show similar sensitivity to metacognitive reading experiences.” Support the claim with evidence or citation**

Authors’ response: Thank you for this suggestion. The information has been supplemented in accordance with the Reviewer's comment.

**6. Line 167 “Motivational” seems not to fit in the sentence perhaps “motivation” could be used.**

Authors’ response: Thank you for this suggestion. The change suggested by the Reviewer was made.

**7. 179 to 182 “Research shows that…” which research? citation**

Authors’ response: Thank you for this suggestion. The information has been supplemented in accordance with the Reviewer's comment.

**8. Line 132 to 142 and Line 179 to 190 are just repetition of same ideas and words.**

Authors’ response: Thank you for this valuable suggestion. We removed the repetitive part from the text.

**9. Why was other aspects of validity analysis not performed or reported to fully validate the scale but only reliability scores. Those analysis could be reported as supplementary materials.**

In the manuscript, we limited ourselves to providing only the reliability indicators of the research tools used for the studied sample, which is consistent with the practice of preparing this type of texts, the main goal of which is not to describe the adaptation of the tools. One of the tools - The Educational Support Questionnaire - has a full description of its validation in the Polish group in a separate article, included in the bibliographic list [67]. In the case of the next tool - Metacognitive Awareness of Reading Strategies Inventory (MARSI) - the adaptation was made for the needs of other studies conducted in a group of Polish children, and the results were published in Dłużniewska A. (2021). *Rozumienie tekstów literackich przez uczniów z uszkodzeniami słuchu*. Kraków: Oficyna Wydawnicza Impuls. A full description of the validation, including content, theoretical and predictive validity of the other two questionnaires (on a larger research sample) was prepared for the needs of separate articles that are currently in the publishing process, which makes it impossible to include them as supplementary material in this text.

**10. Whether analysis on crucial regression assumptions was carried out and met or not was not reported or mentioned to conclude whether the analysis outcome was reliable or not.**

Authors’ response: Thank you for this suggestion. The information has been supplemented in accordance with the Reviewer's comment.

**11. The work could benefit from additional professional editing.**

Authors’ response: We have made every effort to prepare a revised manuscript in accordance with the publishing house's editorial requirements. Attached we also send a certificate of proofreading performed by a native speaker.
